# Accurate Splicing of HDAC6 Pre-mRNA Requires SON

**DOI:** 10.3390/ijms16035886

**Published:** 2015-03-13

**Authors:** Vishnu Priya Battini, Athanasios Bubulya, Paula A. Bubulya

**Affiliations:** Department of Biological Sciences, Wright State University, Dayton, OH 45435, USA; E-Mails: battini.2@wright.edu (V.P.B.); athanasios.bubulya@wright.edu (A.B.)

**Keywords:** pre-mRNA splicing, SON/NREBP, HDAC6, gene expression, BUZ domain, aggresome

## Abstract

Pre-mRNA splicing requires proper splice site selection mediated by many factors including snRNPs and serine-arginine rich (SR) splicing factors. Our lab previously reported that the SR-like protein SON maintains organization of pre-mRNA splicing factors in nuclear speckles as well as splicing of many human transcripts including mRNAs coding for the chromatin-modifying enzymes HDAC6, ADA and SETD8. However, the mechanism by which SON maintains accurate splicing is unknown. To build tools for understanding SON-dependent pre-mRNA splicing, we constructed a minigene reporter plasmid driving expression of the genomic sequence spanning exons 26 through 29 of HDAC6. Following SON depletion, we observed altered splicing of HDAC6 reporter transcripts that showed exclusion of exons 27 and 28, reflecting the splicing patterns of endogenous HDAC6 mRNA. Importantly, loss of HDAC6 biological function was also observed, as indicated by truncated HDAC6 protein and corresponding absence of aggresome assembly activities of HDAC6 binding-of-ubiquitin zinc finger (BUZ) domain. We therefore propose that SON-mediated splicing regulation of HDAC6 is essential for supporting protein degradation pathways that prevent human disease.

## 1. Introduction

Pre-mRNA splicing regulation in eukaryotes is critical for maintaining proper gene expression as well as protein biodiversity. Variation in 5'- and 3'-splice site selection in eukaryotic pre-mRNAs regulates gene expression output by altering the coding sequence of mRNA (reviewed in [1]). Misregulation of pre-mRNA splicing can produce aberrant proteins that disrupt biochemical pathways and/or cell growth controls that contribute to human disease. In recent years, it has become clear that multiple cellular pathways can become simultaneously affected upon altered expression of an individual splicing factor. For example, the SR (serine-arginine-rich)-like protein SON has roles in nuclear organization, splicing regulation, and cell cycle progression (reviewed in [2,3]). SON co-localized with pre-mRNA processing factors in nuclear speckles [4,5,6,7], and was among 33 novel proteins discovered a decade ago during proteomic analysis of purified nuclear speckles [5,8]. Motif analysis revealed several putative functional domains in SON including an RS domain, a glycine-rich patch (G-patch) and double stranded RNA binding domain (DSRBD) [5]. Interestingly, one-third of SON’s amino acid sequence consists of unique repetitive sequence motifs [5] that maintain proper organization of pre-mRNA processing factors in nuclear speckles [6]. Depletion of SON alters organization of splicing factors SRSF1, SRSF2 and U1-70K into doughnut-shaped nuclear speckles [6]. Furthermore, SON colocalized with nascent transcripts at reporter gene loci [9]. RNA fluorescence *in situ* hybridization using splice junction probes demonstrated that nascent transcripts from a stably expressed beta-tropomyosin reporter gene were alternatively spliced in SON-depleted cells, consistent with a function for SON in co-transcriptional pre-mRNA processing [9].

Three independent studies later demonstrated that SON maintains accurate splicing of human mRNAs and pointed toward an important role for SON in regulating the expression of genes involved in cell cycle progression and pluripotency [9,10,11]. SON depletion in human embryonic stem cells (hESCs) results in loss of pluripotency and cell death. Genome-wide RNA profiling in SON-depleted hESCs identified a set of 1994 introns from 1127 genes that had splicing defects following SON depletion, including transcripts from pluripotency genes *OCT4*, *PRDM14*, *E4F1* and *MED24* [11]. Microarray analysis from SON-depleted HeLa cells altered the expression of transcripts that fall into pathways and functional categories such as apoptosis, cell cycle, cancer, DNA replication/recombination/repair, amino acid metabolism, integrin mediated cell adhesion, smooth muscle contraction and G protein signaling [9,10]. Most downregulated transcripts were involved in cell cycle and DNA replication/recombination/repair and upregulated transcripts belonged to the categories of cell signaling, cell death/survival and molecular transport [9,10].

Exon array analysis additionally showed altered splicing (either exon inclusion or exclusion) in 1061 genes and changes in 2067 splicing events, suggesting that SON regulates splice site selection during splicing of many transcripts [9]. Interestingly, intron retention was observed in mRNAs that were downregulated following SON depletion (TUBG1, KATNB1, TUBGCP2, AURKB, PCNT, AKT1, RAD23A and FANCG), indicating that SON is required for accurate intron removal in a subset of transcripts [10]. TUBG1 transcripts showed defective removal of multiple introns, indicating that SON is a coactivator in constitutive splicing. Strengthening weak splice sites by mutagenesis removed transcript dependency on SON for accurate splicing, revealing that SON is required when transcripts have weak splice sites [10]. Furthermore, UV crosslinking and immunoprecipitation (CLIP) studies revealed that SON is physically associated with the mRNAs that were down regulated following SON depletion, suggesting a direct role for SON in splicing. Minigene assays (using the minigene reporter constructs to study intron retention *in vivo*) for TUBG1 pre-mRNA containing exon 7-intron 7-exon 8 cassette or exon 8-intron 8-exon 9 cassette showed that intron removal is directly dependent on the presence of SON [10]. SON RS domain and G-patch were important for proper splicing of TUBG1 pre-mRNA, and the *C*-terminal region containing an RS domain, G-patch and DSRM rescued splicing to a significant extent [10].

## 2. Results and Discussion

### 2.1. SON Is Required for Retention of Exons 27 and 28 in Endogenous HDAC6 Transcripts and Exogenous HDAC6 Minigene Reporter Transcripts

**Figure 1:**
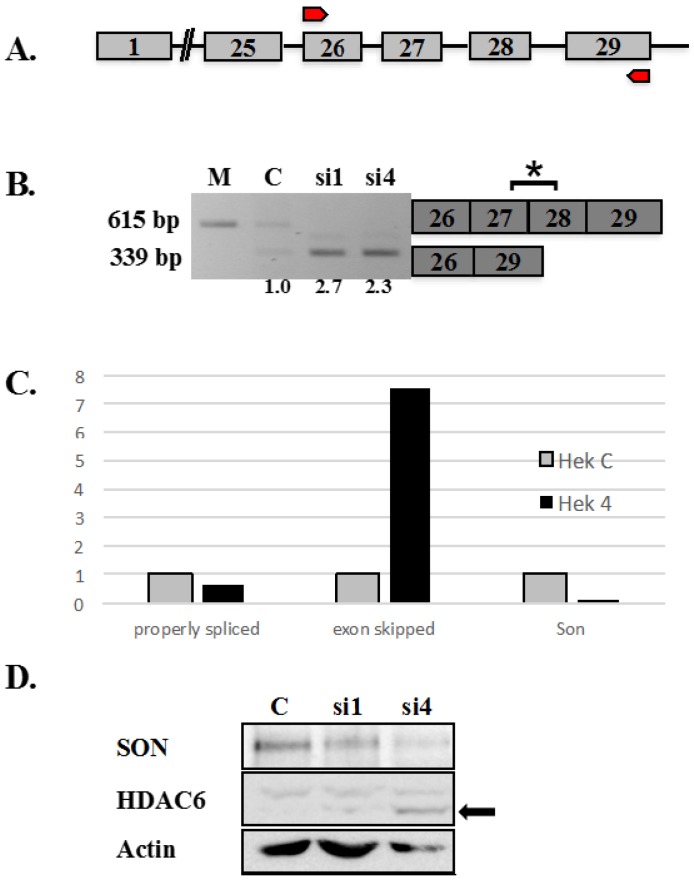
Altered splicing of HDAC6 pre-mRNA in SON depleted HeLa cells. (**A**) Human HDAC6 contains 29 exons represented as boxes and the intervening introns represented as lines joining exons. Red arrows represent the region of genomic DNA used to construct HDAC6 minigene reporter plasmid shown in Figure 2, Figure 3 and Figure 4; (**B**) RT-PCR performed using primers that amplify endogenous HDAC6 transcripts shows exon skipping in SON siRNA-treated samples compared to mock (M) and control (C). Boxes to the right indicate the region of HDAC6 transcripts detected by RT-PCR. Asterisk indicates exons 27 and 28 are skipped in SON-depleted cells. Numerical values below lanes represent the ratio of transcripts having retained:excluded introns; (**C**) Human embryonic kidney cells treated with either control siRNAs or si4 siRNA duplex targeting SON (Hek 4) were used for qRT-PCR with HDAC6 exon-junction oligos. SON depleted cells showed significantly reduced expression of SON transcripts (Son) as well as reduction of properly spliced HDAC6 transcripts, but showed increased skipping of exons 27 and 28 (exon skipped) for endogenous HDAC6 transcripts; and (**D**) HeLa cells were transfected with control siRNA oligo (C) and two different siRNA oligos targeting SON. Forty-eight hours post-transfection, protein was extracted and immunoblotting was performed with antibodies against SON (WU09), HDAC6 and actin. Western blot confirmed reduced SON protein in cells treated with siRNA duplexes targeting SON. Arrow indicates that the molecular mass of HDAC6 is 10 kDa smaller following treatment with siRNA targeting SON compared to control siRNA treated cells.

Reducing SON expression level affected all different types of splicing, including exon skipping [9,10,11]. SON depletion resulted in skipping of exon 6 in rat beta-tropomyosin (BTM) minigene reporter transcripts. Misregulated splicing was also shown in a subset of human transcripts including skipping of exons 27 and 28 in endogenous HDAC6 transcripts ([9] and Figure 1B). Histone deacetylases (HDACs) remove acetyl groups from nucleosomal histones at conserved lysine residues, as a critical step of controlling chromatin accessibility and transcription activity during gene regulation. HDACs can also deacetylate lysine residues of transcription factors and thereby regulate their DNA binding ability. HDAC6 is a class IIB histone deacetylase and is a cytoplasmic protein (reviewed in [12]). HDAC6 is the only histone deacetylase that contains two functional catalytic (CAT) domains and a *C*-terminal BUZ (binding-of-ubiquitin zinc finger) domain [13]. The two catalytic histone deacetylase domains function in deacetylating α-tubulin, playing a key role in cell motility [14]. The HDAC6 BUZ-domain (amino acids 1134–1192) is encoded by 174 nucleotides contained within exons 27 and 28 of HDAC6 mRNA.

We tested whether SON depletion that results in skipping exons 27 and 28 in HDAC transcripts would ultimately interfere with BUZ domain functions. Gene structure of HDAC6 is shown in Figure 1A, indicating exons as numbered boxes, connected by lines that represent introns. Transcripts including sequence spanning from the beginning of exon 26 through the end of exon 29 (indicated by red arrows in Figure 1A) are the focus of our current study. As shown previously, exons 27 and 28 of endogenous HDAC6 transcripts were skipped in SON-depleted cells but not in cells treated with control siRNAs (Figure 1B and [9]). Importantly, we also detected skipping of exons 27 and 28 in HDAC6 transcripts in human embryonic kidney cells (Figure 1C). Our studies did not determine whether altered splicing of HDAC6 transcripts affects HDAC6 protein expression or HDAC6 function. To test this, we examined the effects of SON depletion on HDAC6 protein. Immunoblot data in Figure 1 demonstrates that endogenous HDAC6 protein has a molecular mass roughly 10 kDa smaller in extracts from SON-depleted HeLa cells compared to HDAC6 in extracts from controls (Figure 1D); this truncated protein is predicted to not contain the BUZ domain due to absence of exons 27 and 28 in the mature HDAC6 mRNA under these conditions. This result suggests a biological relevance for SON for maintaining HDAC6 functions in human cells. We next compared patterns of exon skipping for endogenous *versus* reporter HDAC6 transcripts. Notably, incorrectly spliced HDAC6 transcripts were evident in samples having partial SON reduction, indicating that reduced SON expression is adequate for disrupting splicing of HDAC6 transcripts.

**Figure 2:**
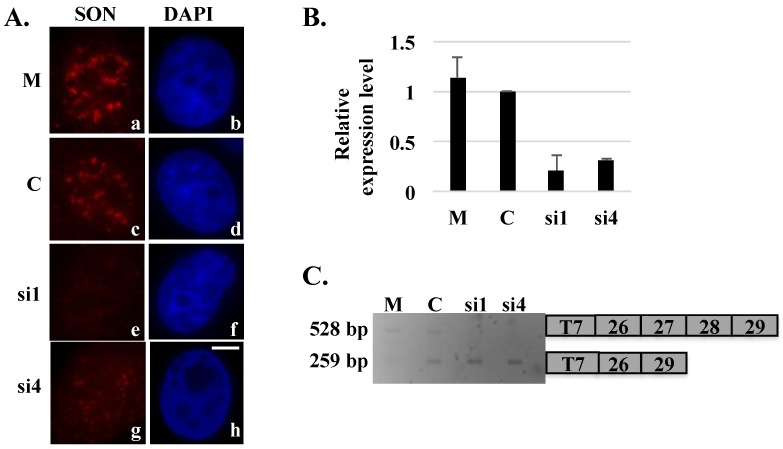
Skipping of exons 27 and 28 of the HDAC6 reporter minigene in SON-depleted cells. HeLa cells transiently transfected with HDAC6 reporter plasmid and treated with mock (M), control (C) or SON siRNA duplexes were either processed for immunofluorescence (**A**) or harvested to perform qRT-PCR (**B**) to show SON depletion. Cells treated with SON siRNAs and immunolabeled with anti-SON antibodies in (**A**) do not show SON labeling in nuclear speckles (**e** and **g**) when compared to mock treated (**a**) or control siRNA-treated (**c**) cells. DNA was stained with DAPI (**b**, **d**, **f** and **h**). Scale bar = 5 µm. qRT-PCR in (**B**) showed reduction of SON mRNA levels in SON siRNA-treated samples compared to mock and control siRNA treated samples; (**C**) RT-PCR performed using primers that specifically target reporter minigene transcripts shows exon skipping in SON siRNA-treated samples.

Reporter minigenes typically contain a segment of genomic DNA corresponding to regions of a gene that become alternatively spliced (or mis-spliced) under a specific condition. When expressed in cells, these minigene reporters allow in *in vivo* analysis of *cis*-regulatory elements (such as cryptic splice sites, exonic splicing enhancers and exonic splicing silencers) and *trans*-acting factors such as splicing factors and splicing repressors [15]. To examine splicing changes in exogenous HDAC6 transcripts upon SON depletion, HeLa cells transiently transfected with plasmid expressing our human HDAC6 reporter minigene, were treated with siRNA oligos targeting SON mRNA. Reduction of SON transcripts was confirmed by qRT-PCR (Figure 2B). SON-depleted cells showed reduced immunolabeling of SON in nuclear speckles compared control siRNA-treated cells (Figure 2A; Sharma *et al.*, 2010 [9]). Polyacrylamide gel electrophoresis of RT-PCR products indicated that properly spliced HDAC6 transcripts containing both exons 27 and 28 were detected in control cells, while exons 27 and 28 were predominantly skipped in HDAC6 transcripts from SON-depleted cells, and properly spliced transcripts were not detected (Figure 2C).

**Figure 3:**
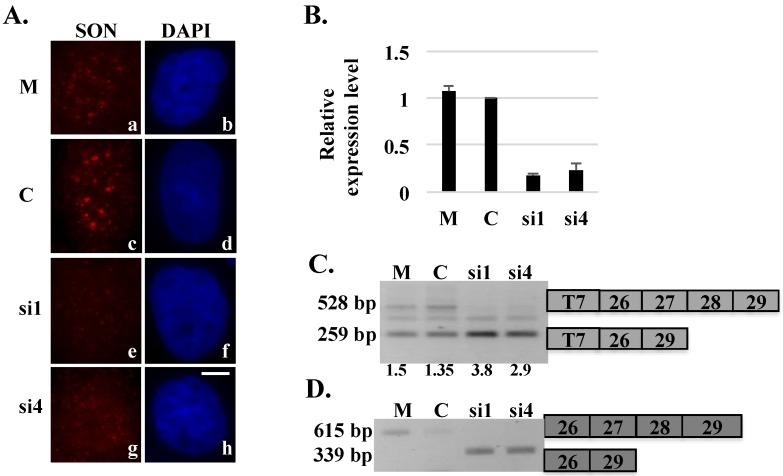
Stably expressed HDAC6 reporter transcripts show exon skipping after SON depletion. HeLa HDAC6 clone 69 stably expressing HDAC6 reporter plasmid was transfected with mock (M), control (C) or SON siRNA duplexes and processed for either immunofluorescence (**A**) or harvested to perform qRT-PCR (**B**) to show SON depletion. Cells treated with SON siRNA and immunolabeled with anti-SON antibodies in (**A**) do not show SON labeling in nuclear speckles (**e** and **g**) when compared to mock (**a**) or control siRNA (**c**) treated cells. DNA was stained with DAPI (**b**, **d**, **f** and **h**). Scale bar = 5 µm. qRT-PCR in (**B**) showed reduction of SON mRNA levels in SON siRNA-treated samples when compared to mock and control siRNA treated samples; (**C**) RT-PCR performed using primers that specifically target exogenous reporter HDAC6 minigene transcripts shows exon skipping in SON siRNA-treated samples; and (**D**) RT-PCR performed with primers that specifically target endogenous HDAC6 transcripts shows exon skipping in SON siRNA-treated samples. Numerical values below lanes in (**C**) represent the ratio of transcripts having retained:excluded introns.

To further investigate SON-mediated splicing of HDAC6 transcripts, we constructed HeLa cell lines stably expressing our HDAC6 reporter minigene. Three HeLa HDAC6 clones stably expressing the HDAC6 reporter minigene were transfected with SON siRNA to test splicing patterns of HDAC6 minigene reporter transcripts. SON depletion was performed in three separate HeLa HDAC6 stable cell lines that each consistently showed skipping of exons 27 and 28 both in exogenous HDAC6 transcripts (Figure 3C, shows one representative data set for clone 69) and in endogenous HDAC6 transcripts (Figure 3D). The rationale for testing multiple clones was based upon our hypothesis that independent clones that presumably contain HDAC6 minigene integrated randomly at different genomic locations may yield different splicing results if chromatin context influences splicing of HDAC6 transcripts. However, the similarity of results among multiple independent clones indicates that chromatin context probably does not influence splicing of these specific exons.

### 2.2. Aberrant Splicing of HDAC6 in Absence of SON Is Biologically Significant

**Figure 4:**
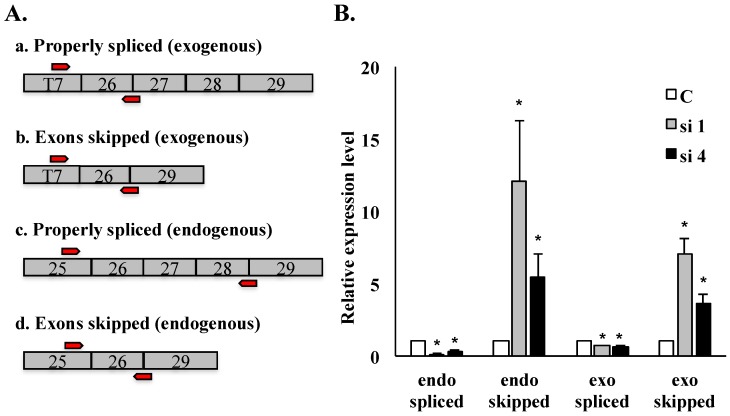
qRT-PCR analysis of HDAC6 exon skipping in SON-depleted cells. qRT-PCR performed on HeLa HDAC6 stable cells post-SON depletion. (**A**) Four sets of exon junction primers indicated by red arrows (see Experimental Section) were used to amplify splice variants of exogenous reporter transcripts as well as of endogenous HDAC6 transcripts; (**B**) Relative transcript level for properly spliced *versus* exon skipped transcripts is measured both for endogenous and for exogenous HDAC6 transcripts. Error bars were calculated by measuring standard error from three individual experiments (* *p* ≤ 0.05).

We performed quantitative RT-PCR to examine the abundance of properly spliced *versus* mis-spliced HDAC6 transcripts in SON-depleted cells compared to controls. Primers spanning exon junctions specifically amplified properly spliced *versus* mis-spliced transcripts, while primers targeted specifically to sequences upstream of exon 26 allowed detection of exogenous or endogenous HDAC6 transcripts as shown in Figure 4A. qRT-PCR was performed in the SON-depleted HeLa HDAC6 stable cell line “clone 69”, and SON depletion was validated by qRT-PCR. In SON-depleted cells, the level of properly spliced HDAC6 transcripts was reduced (for both endogenous and exogenous HDAC6 transcripts) while the level of mis-spliced transcripts increased in comparison with respective transcript levels in cells treated with control siRNA (Figure 4B). This data shows that mis-splicing of HDAC6 transcripts is more prevalent in SON-depleted cells, and it indicates the efficacy of our HDAC6 minigene reporter system for mimicking misregulated HDAC6 pre-mRNA splicing patterns.

Next, we performed *in situ* analysis to test whether biological function of HDAC6 was disrupted in SON-depleted cells. An aggresome assembly assay determined that HDAC6 protein expressed in SON-depleted cells lacks normal BUZ domain function. Under normal cellular conditions, the HDAC6 BUZ domain associates with polyubiquitin and thus with ubiquitinated misfolded proteins [16]. Formation of misfolded proteins increases under stress, and this condition can become toxic [17]. In several human disorders, the accumulation of misfolded proteins leads to cell death. One mechanism of misfolded protein degradation involves HDAC6-dependent formation of aggresomes [16]. HDAC6 interacts with dynein motor proteins as well as ubiquitinated misfolded proteins and directs them via microtubules to the microtubule-organizing center (MTOC), where aggresomes assemble [16] and misfolded proteins are degraded by local proteases and autophagy proteins. Mutant HDAC6 lacking either of the catalytic deacetylase domains or the BUZ domain fails to support aggresome assembly, demonstrating the importance of these HDAC6 domains in aggresome function [16].

**Figure 5:**
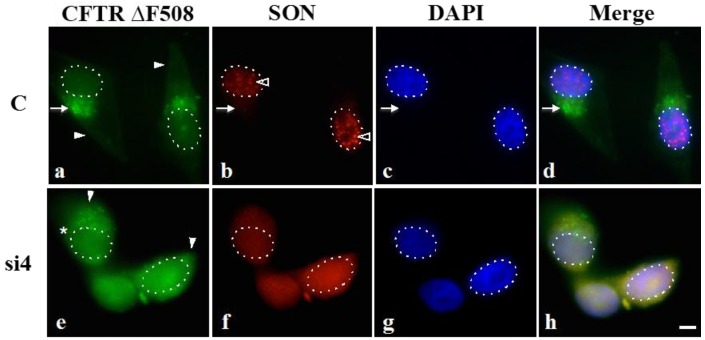
SON depletion reduces HDAC6-mediated aggresome formation. HeLa cells transiently expressing GFP-CFTR ∆F508 were transfected with either control siRNA duplexes (C) or SON siRNA duplexes and then treated with proteasome inhibitor MG132. Immunofluorescence labeling with anti-SON WU14 antibody indicates that SON normally localizes to nuclear speckles (open arrowheads in **b**) and that SON labeling is reduced in nuclear speckles of cells treated with SON siRNA duplexes (**f**). DAPI was used to stain DNA (**c** and **g**) and used to highlight the nuclear boundary in all panels (dotted lines). Arrow in (**a**–**d**) indicates GFP-CFTR ∆F508 localized at aggresomes. Asterisk in (**e**) marks a SON-depleted cell that retains a few cytoplasmic foci of GFP-CFTR ∆F508 (see Discussion). Filled arrowheads mark GFP-CFTR signal that is more diffuse in the cytoplasm of SON-depleted cells (**e**) while it is predominantly concentrated at aggresomes in controls (**a**). Triple merge shown in (**d**) and (**h**). Scale bar = 5 µm.

To analyze HDAC6 function in SON depleted cells, we exogenously expressed GFP-CFTR ∆F508 (a straightforward approach to produce fluorescently labeled misfolded protein in cells) and then induced aggresome formation *in situ*. These cells were then transfected with SON siRNA, and allowed time for SON depletion before aggresome formation was induced. Cells treated with control siRNA showed robust signal for GFP-CFTR ∆F508 in large clusters of cytoplasmic aggresomes (Figure 5a,d, arrow), while diffuse cytoplasmic GFP signal was very low (Figure 5a, filled arrowheads mark cytosol). In cells treated with SON siRNA, misfolded protein was predominantly cytoplasmic and diffuse (marked by filled arrowheads in Figure 5e). An occasional SON-depleted cell showed small foci of GFP-CFTR ∆F508 (indicated by an asterisk in Figure 5e) that could be explained by the low amount of properly spliced transcript detected in SON depleted cells that would produce “wild type” HDAC6 that may contribute to some minimal level of aggresome assembly. This is not unexpected, since the overall goal of this experiment was to see that in the majority of SON-depleted cells, the GFP-CFTR ∆F508 signal should be more dispersed due to the expression of truncated HDAC6 protein. Our results suggest that HDAC6 protein lacking the BUZ domain reduces the extent of aggresome assembly. Moreover, our results are consistent with data reported by Kawaguchi *et al.*, (2003) [16] in that the HDAC6 protein lacking BUZ domain fails to support aggresome assembly.

## 3. Experimental Section

### 3.1. HDAC6 Reporter Minigene Construction

BAC clone RP11-416B14 containing the human HDAC6 gene was purchased from Children’s Hospital Oakland Research Institute (CHORI; Oakland, CA, USA). HDAC6 genomic sequence spanning exon 26 through 29 was PCR amplified by using forward primer 5'-GGGGGATCCGGGGCCTCAGAATCTCAG-3' containing a flanking BamH1 restriction site (underlined) and reverse primer 5'-GGGGCTCGAGAACAGCTTGTACTTTATT-3' containing a flanking Xho1 restriction site (underlined). This HDAC6 “minigene” was then cloned into pcDNA3.1-V5-His-A (Life Technologies, Grand Island, NY, USA). DNA sequencing confirmed that no mutations were present in the HDAC6 reporter gene construct.

### 3.2. Cell Culture, Transfections and RNAi

HeLa cells (a gift from D. Spector, Cold Spring Harbor Laboratory, Cold Spring Harbor, NY, USA) were grown and maintained in 100 mm × 20 mm culture dishes in Dulbecco’s Modified Eagle Medium (DMEM; Hyclone, Thermo Scientific, Waltham, MA, USA) with 10% fetal bovine serum (FBS; Hyclone, Thermo Scientific) and 1% penicillin/streptomycin (Invitrogen). All cells were maintained in a humidified incubator (37 °C) in the presence of 5% carbon dioxide. For transfections, 40 µg of salmon sperm DNA, 2 µg of HDAC6 reporter plasmid or GFP-CFTR ∆F508 plasmid and 250 µL of cell suspension (containing ~2.5 × 10^7^ cells) in a 4 mm gap cuvette. Electroporation was performed by using GenePulser × Cell (250 V, 950 μF and 4 Ω; BioRad, Hercules, CA, USA). To construct HeLa HDAC6 stable cell lines, HeLa cells were transfected with HDAC6 reporter minigene plasmid as described above, and positive clones were selected by adding G418 (1 mg/mL) to the culture medium. For primary screening of clones stably expressing HDAC reporter transcripts, RNA was extracted from the 96 clones followed by DNase treatment and positive clones were selected by RT-PCR analysis using T7 forward primer and E29 reverse primer. SON depletion was achieved by RNAi using SON siRNA duplexes (si1 catalog # J-012983-05 or si4 J-012983-08; Thermo Scientific) for 48 h. A non-targeting siRNA duplex for luciferase (catalog No. D-001210-02; Dharmacon RNA Technologies) was used as a control to ensure that the changes observed are not simply due to activation of RNAi machinery. SON depletion was confirmed by immunofluorescence with antibodies against SON (WU14), qRT-PCR using primer sets targeting SON mRNA, or by immunoblotting with antibody WU09. To test endogenous HDAC6 activity, cells transfected with GFP-CFTR ∆F508 were incubated for 24 h prior to treatment with siRNA duplexes targeting SON, incubated an additional 32 h, then treated with MG1232 (10 mM) for 16 h prior to SON immunolabeling as described above.

### 3.3. Immunofluorescence

Cells grown on coverslips were washed once with 1× PBS and fixed with 2% formaldehyde (in 1× PBS) for 15 min followed by three washes with 1× PBS for 5 min each. The cells were then permeabilized for 5 min with 0.2% TritonX-100 and washed three times with 0.5% normal goat serum (NGS, Thermo Scientific) in 1× PBS. The coverslips were then incubated with primary antibodies diluted in 0.5% NGS-1X PBS cell side up in a humidified chamber for one hour at room temperature. Rabbit polyclonal antibodies were developed (Covance; Denver, PA, USA) against the *C*-terminal peptide sequence SPNKKHAKATAATV near the *C*-terminus of SON (WU13; 1:100), and against the *N*-terminal peptide sequence CEESESKTKSH near the *N*-terminus of SON (WU14; 1:1000) and monoclonal anti-SF2/ASF AK103 (1:2500; provided by A. Krainer, Cold Spring Harbor Laboratory, Cold Spring Harbor, NY, USA). Coverslips were washed three times with 0.5% NGS in 1× PBS and incubated for one hour with donkey anti-mouse (Cy5) and donkey anti-rabbit (FITC or TxRed) antibodies (1:500; Jackson ImmunoResearch Laboratories, West Grove, PA, USA), then washed two times with 0.5% NGS in 1× PBS for 5 min each. To stain DNA, coverslips were incubated for 5 min in 4',6-diamidino-2-phenylindole (DAPI; 10 μg/mL diluted 1:1000 in 1× PBS), washed once with 1× PBS. Coverslips were mounted in anti-fade mounting medium (*p*-phenylenediamine, glycerol, pH 8.0–9.0) or *N*-propyl-gallate mounting medium (for CY5-conjugated antibodies). The slides were imaged using DeltaVision RT microscope equipped with a 60× objective (1.4 numerical aperture; Olympus, Tokyo, Japan), and softWoRx 2.50 software (DeltaVision by Applied Precision/GE Healthcare, Issaquah, WA, USA). Raw images were displayed as volume projections.

### 3.4. Cell Extract Preparation and Immunoblotting

Cells were washed briefly with 1× PBS and scraped in 1× PBS and collected into tubes. The cells were then resuspended in 1× Laemmli buffer (0.01 M Tris (pH 6.8), 1% SDS, 10% glycerol and 0.1% β-mercapthoethanol). For immunoblotting, equal amounts of protein was loaded to 7% SDS-PAGE and transferred to a nitrocellulose membrane. The membrane was then blocked with 5% not-fat dry milk in PBST. Anti-SON (Wu09, 1:1000, a polyclonal antibody directed against the peptide MDSQMLATSS repeats) or anti-β-actin (1:3000, Cat. No. A5441; Sigma-Aldrich, St. Louis, MO, USA) were used as primary antibodies. For secondary antibodies, horseradish peroxidase (HRP)-conjugated donkey anti-mouse or HRP-conjugated donkey anti-rabbit IgG (Jackson ImmunoResearch Laboratories, Cat. Nos. 711-035-152 and 715-035-150), were used at 1:25,000. ECL Western blotting substrate (Thermo Scientific, Cat. No. 32106) was used to detect HRP.

### 3.5. RNA Extraction and DNase Treatment

RNA extraction was performed by using Qiagen RNAeasy mini prep kit according to manufacturer instructions (Valencia, CA, USA). RNA was eluted in 30 µL RNase-free water and stored at −80 °C. Turbo DNase activation buffer (10%), 5 µg of RNA, and Turbo DNase (1 µL) were mixed and brought to 50 µL final volume with RNase free water. The samples were incubated at 37 °C for 30 min followed by the addition of DNase inactivation reagent (10%) and incubated for 2 min at room temperature with occasional mixing. The samples were centrifuged for 1.5 min at 10,000× *g* and the supernatant containing DNA-free RNA was transferred to fresh 1.5 mL tubes and stored at −80 °C.

### 3.6. RT-PCR and Quantitative RT-PCR

For Figure 2, RT-PCR was performed using qScript-One step RT-PCR system (Cat. No. 95047; Quanta Biosciences, Gaithersburg, MD, USA). For one-step RT-PCR, 100 ng of DNase treated RNA was used as template along with 3 µM forward and reverse primers, 2× SYBRgreen buffer, 1 µL reverse transcriptase and RNase-free water in 10 µL final volume. Amplification was performed for 40 cycles. As exon skipping occurs in HDAC6 transcripts upon SON depletion, reverse transcription-PCR reactions amplify more than one PCR product when Ex25-Ex29 or T7-Ex29 primer pairs were used. In these cases, the RT-PCR products were applied to 2% agarose gels to assess relative abundance of different splice forms. Imaging was performed using Foto/UV 21 (Fotodyne Inc., Hartland, WI, USA). For Figure 3, RT-PCR was performed using qScript-two step RT-PCR system (Quanta Biosciences, MD Cat. No. 95073); cDNA was prepared by mixing 4 µL cDNA fast mix, 500 ng DNase-treated RNA, 1 µL reverse transcriptase and RNase-free water. The samples were incubated in DNA engine DYAD thermo cycler (MJ Research, Waltham, MA, USA) with cycle conditions: 5 min at 22 °C; 30 min at 42 °C and 5 min at 85 °C. cDNA was either stored at −20 °C or diluted three fold and used as template for qPCR. For qPCR, master mixes were prepared separately for each primer set. Five microliters of PerfecTa fast mix, 3 µM forward primer, 3 µM reverse primer and RNase-free water were mixed per reaction to make master mix and aliquoted to the reaction wells followed by addition of 3 µL of diluted cDNA template. The quantitative results (*C*_t_ values) of GAPDH qRT-PCR were subtracted from SON qRT-PCR results to determine Δ*C*_t_. ΔΔ*C*_t_ was calculated by subtracting the effect of control siRNA. Fold change was determined by calculating 2^−ΔΔ*C*t^. A graph was then plotted between the average fold change from three biological replicates and samples. Standard error was calculated for fold change from three biological replicates. Product was not detected when reverse transcriptase was omitted (not shown).

### 3.7. Primer and Probe Design for Quantitative RT-PCR Analysis of HDAC6 Transcripts

An oligo complementary to sequences in exon 25 was used to specifically detect endogenous HDAC6 transcripts (as the HDAC6 minigene reporter does not contain exon 25), while an oligo complementary to sequences near the T7 promoter region of the HDAC6 minigene was used to specifically detect exogenous HDAC6 transcripts (as the T7 sequences are absent in endogenous HDAC6 transcripts). HDAC6 minigene transcripts were detected with an exon junction oligo having reverse complementary sequence to the last 12 nt (nucleotides) of exon 26 plus the first 12 nt of exon 27, while mis-spliced HDAC6 transcripts were detected with an oligo having reverse complementary sequence to the last 12 nt of exon 26 and the first 12 nt of exon 29.

## 4. Conclusions

To gain insight into splicing functions for SON, we relied on reporter minigene containing the genomic DNA spanning exons 26 through 29 of HDAC6 and observed that exons 27 and 28 were excluded in both endogenous and exogenous HDAC6 transcripts indicating the presence of all the cis-elements required for SON-regulated splicing of these exons. Since results obtained from transient minigene expression and from multiple independent stable clones expressing reporter transcripts were all consistent, one important conclusion that can be drawn from these observations is that there is probably no connection between chromatin context and splicing regulation by SON for splicing of exons 27 and 28 of HDAC6 transcripts. Finally, we have shown evidence that splicing regulation of HDAC6 pre-mRNA is a critical step for ensuring expression of functional HDAC6 protein in human cells.

Understanding the mechanism by which SON regulates splicing events of several selected genes will require future experiments that define *cis*-acting elements (ESEs/ISEs) in transcripts that interact with SON for accurate splicing, as well as the domains of SON that mediate splicing activities. ESE finder predicts weak splice sites at the 5'-splice site of exon 27 and at the 3'-splice site of exon 28 (data not shown) that is consistent with data reported in other studies, and that possibly explains why HDAC6 splicing is dependent on presence of SON [10]. Future experiments will be required to validate these splice sites in HDAC6 as “weak” and to demonstrate that strengthening these sites might alter their dependence on SON for accurate splicing.

Moreover, SON depletion causes exclusion of exons 27 and 28 in HDAC6 transcripts that code for the BUZ domain of HDAC6 protein. To our knowledge, there is no evidence of naturally occurring HDAC transcripts lacking exons 27 and 28 reported to date. However, it is reasonable to consider that genetic mutations disrupting SON expression and/or function in humans could produce HDAC6 protein lacking the BUZ domain. Polyubiquitinated misfolded protein transport to aggresomes by dynein motor proteins is mediated by HDAC6 via its association with dynein motor proteins through a dynein-motor binding (DMB) domain located between the two deacetylase domains. Association with polyubiquitinated misfolded proteins occurs through the HDAC6 BUZ domain. Thus, HDAC6 helps link misfolded proteins to dynein motor protein complexes for transport to aggresomes [16,18]. Disrupted binding of HDAC6 to ubiquitin would thus interfere with recruitment of misfolded proteins to aggresomes [16]. The minigene reporter tools we report here to evaluate HDAC6 expression will be useful in the future to measure SON activity in cells of patients with mutations in the *SON* gene. Our reporter system will be useful to tease out the mechanisms of SON-mediated splicing of HDAC6 transcripts, and can perhaps be used in the future for developing new methods to restore HDAC6 function *in vivo*. Since failure to degrade misfolded proteins results in cell death, approaches that restore HDAC6 splicing may be key for therapeutic intervention in diseases such as cancer [19] as well as for neurodegenerative diseases [18].
